# Selectins and Immune Cells in Acute Myocardial Infarction and Post-infarction Ventricular Remodeling: Pathophysiology and Novel Treatments

**DOI:** 10.3389/fimmu.2019.00300

**Published:** 2019-02-27

**Authors:** Brian R. Weil, Sriram Neelamegham

**Affiliations:** ^1^Department of Physiology and Biophysics, University at Buffalo, State University of New York, Buffalo, NY, United States; ^2^Department of Medicine, University at Buffalo, State University of New York, Buffalo, NY, United States; ^3^Department of Chemical & Biological Engineering, University at Buffalo, State University of New York, Buffalo, NY, United States

**Keywords:** glycan, selectin (sE, sL, sP-selectin), leukocyte-endothelial cell adhesion, heart disease, myocardial infarction, post-infarct repair, stem cells, mesenchymal (stromal) stem cells

## Abstract

The glycosciences aim to understand the impact of extracellular and intracellular carbohydrate structures on biological function. These glycans primarily fall into three major groups: lipid-linked carbohydrates that are referred to as glycosphingolipids or simply glycolipids; relatively short carbohydrate chains that are often O- or N-linked to proteins yielding common glycoproteins; and extended linear polymeric carbohydrate structures that are referred to as glycosaminoglycans (GAGs). Whereas, the impact of such carbohydrate structures has been extensively examined in cancer biology, their role in acute and chronic heart disease is less studied. In this context, a growing body of evidence indicates that glycans play an important role in immune mediated cell recruitment to damaged heart tissue to initiate wound healing and repair after injury. This is particularly important following ischemia and reperfusion that occurs in the heart in the setting of acute myocardial infarction. Here, immune system-mediated repair of the damaged myocardium plays a critical role in determining post-infarction ventricular remodeling, cardiac function, and patient outcome. Further, alterations in immune cell activity can promote the development of heart failure. The present review summarizes our current understanding of the phases of immune-mediated repair following myocardial infarction. It discusses what is known regarding glycans in mediating the recruitment of circulating immune cells during the early inflammatory stage of post-infarction repair, with focus on the selectin family of adhesion molecules. It offers future directions for research aimed at utilizing our knowledge of mechanisms underlying immune cell recruitment to either modulate leukocyte recruitment to the injured tissue or enhance the targeted delivery of biologic therapeutics such as stem cells in an attempt to promote repair of the damaged heart.

## Introduction

Cardiovascular diseases, including atherosclerosis, myocardial infarction (MI), and heart failure, represent a primary cause of morbidity and mortality in Western civilization and are rapidly becoming a major epidemic in developing and underdeveloped nations. While the use of lipid-lowering statins, angiotensin-converting-enzyme inhibitors, and medical devices (e.g., coronary stenting, defibrillators, and ventricular assist devices) have reduced the incidence of death, survivors of primary MI are susceptible to secondary heart failure and reinfarction. The factors governing patient outcome are complex, but generally driven by metabolic changes (1), the acute phase response (2), and alterations in leukocyte migration patterns (3). While various basic science studies have examined the putative role of glycosylation in aspects related to cell adhesion and cell signaling, an integrated understanding of their potential impact on the progression of cardiovascular diseases is lacking. The current review addresses this topic with a focus on the impact of selectins in regulating immune system-mediated cardiac repair following myocardial infarction, the current status of anti-selectin therapies directed to the heart, and novel regenerative therapeutic approaches that attempt to exploit naturally occurring cell adhesion processes to improve patient outcome. The discussion suggests that instead of completely abolishing all immune cell interactions following ischemia-reperfusion, a more nuanced approach that finely modulates the relative contributions of different leukocyte populations and exploits glycan-mediated stem cell delivery may be more beneficial.

## Pathophysiology of Acute Myocardial Infarction

Acute MI is typically caused by the abrupt interruption of blood flow through an epicardial coronary artery by plaque rupture and the subsequent formation of an occlusive thrombus, which leads to cardiac myocyte death and compromised heart function. Although the implementation of timely reperfusion strategies has reduced the acute mortality associated with MI, improved patient survival has increased the incidence of chronic heart failure, due in large part to adverse remodeling of the damaged left ventricle (LV) following the initial ischemic event (4). Thus, despite surviving an initial MI, many patients experience a dramatic deterioration in quality of life with the onset of heart failure, a condition for which there is currently a paucity of treatment options that address the fundamental problem of cardiomyocyte loss. This paradigm shift has re-directed translational research efforts toward investigation of the downstream consequences of MI in hopes of identifying novel approaches to reduce adverse LV remodeling and prevent the onset of heart failure.

**Figure 1:**
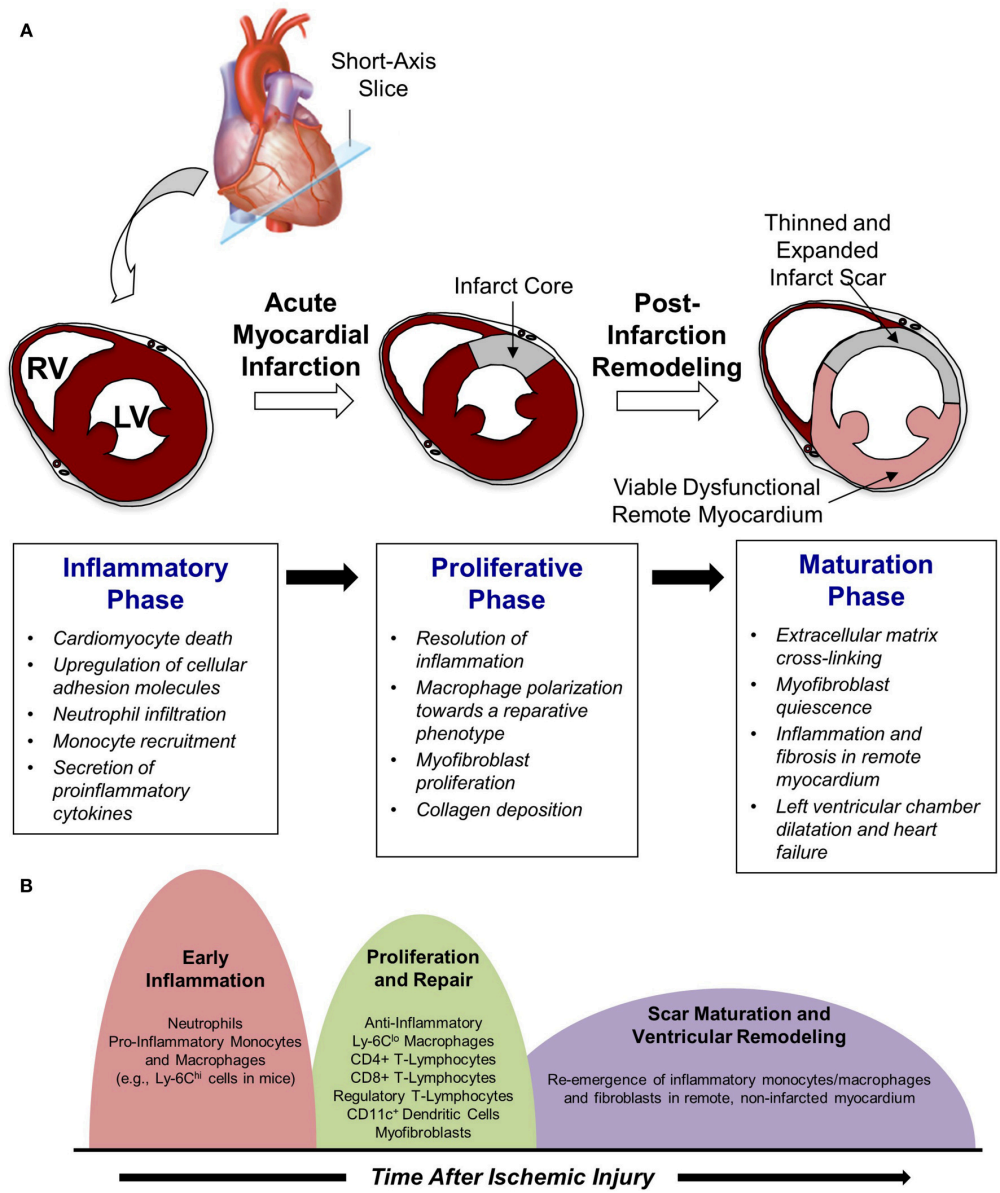
Cardiac repair after myocardial infarction. **(A)** The three phases of repair. **(B)** Temporal changes in leukocyte populations during repair phase.

Work in this area has demonstrated that cardiac repair after MI is characterized by a series of time-dependent events orchestrated by the innate immune system (Figure 1A). This begins immediately after the onset of necrotic cell death with intense sterile inflammation and myocardial infiltration of a variety of immune cell subtypes including neutrophils and monocytes during the first several days after MI (Figure 1B) (5). Subsequently, there is a transition to a reparative and proliferative phase in which inflammation is resolved, myofibroblasts proliferate, and collagen deposition leads to scar formation. Finally, the scar undergoes a maturation process characterized by extracellular matrix (ECM) cross-linking and quiescence of myofibroblasts. A proper balance and timely resolution of the inflammatory, proliferative, and maturation phases of repair are essential to produce an appropriate wound healing response. For example, an inflammatory phase of excessive magnitude or duration can exacerbate tissue damage, impair scar formation, and perpetuate further cardiac myocyte loss, thereby promoting adverse LV remodeling characterized by infarct expansion, chamber dilatation, and contractile dysfunction (6). Indeed, several experimental (3, 7, 8) and clinical (9–11) studies demonstrate that excessive mobilization and/or recruitment of inflammatory cells impair post-MI healing and is associated with adverse outcomes. Thus, investigation of biological mechanisms underlying the inflammatory, proliferative, and maturation phases of cardiac repair has intensified with the hope that improved understanding of these processes may facilitate the development of therapeutic strategies that optimize healing of the damaged heart following MI. Glycans are an integral part of such studies due to their critical role on leukocyte-endothelial cell adhesion mechanisms and their value as biomarkers of metabolic alteration. The following text discusses in detail the three remodeling phases following acute MI.

### Inflammatory Phase of Post-infarction Repair

Although a variety of cell types are involved in mediating post-MI inflammation, circulating leukocytes play a particularly prominent role. Prolonged ischemia and reperfusion injury elicits cardiomyocyte death, primarily via necrosis but also through apoptosis and autophagy (12). Myocyte death, as well as damage to the ECM, prompts the release of danger-associated molecular patterns (DAMPs) that attract circulating immune cells via binding to pattern recognition receptors (PRRs). Besides passive release from necrotic myocytes and damaged ECM, DAMPs and pro-inflammatory cytokines may also be secreted from stressed or reversibly injured myocytes surrounding the infarct core to initiate recruitment of circulating granulocytes and monocytes. These signals also elicit endothelial activation and rapid upregulation of cellular adhesion molecules such as P- and E-selectin that facilitate leukocyte adhesion, endothelial rolling, and, ultimately, extravasation into damaged tissue.

Neutrophils are the first immune cell type to infiltrate the infarcted myocardium and do so via expression of selectin ligands that initiate adhesion to activated endothelial cells. Slow rolling along the endothelial surface allows neutrophils to sense chemokines bound to glycosaminoglycans, subsequently promoting integrin activation, firm adhesion, and transmigration at endothelial junctions. Extravasated neutrophils phagocytize cellular debris, release proteolytic enzymes, and generate reactive oxygen species to degrade extracellular matrix and initiate the wound healing response (13). However, these actions perpetuate further inflammation and can exert direct cytotoxic effects on viable myocytes and blood vessels, thereby exacerbating myocardial damage associated with reperfusion injury (14). Excessive intravascular neutrophil accumulation also promotes capillary damage and microvascular plugging that can result in microvascular obstruction and the “no-reflow” phenomenon, thereby compromising the quality of reperfusion and extending the duration of ischemic injury (15). Moreover, neutrophil-mediated endothelial cell injury promotes the development of interstitial edema, which can further impair microvascular perfusion via extravascular compression (16). The adverse effects of neutrophil infiltration can extend beyond the early post-reperfusion period as well, as disproportionate neutrophil accumulation may interfere with the recruitment of additional leukocyte populations and the transition from the inflammatory to proliferative phase of cardiac repair, an essential component of tissue healing. Nevertheless, experimental neutrophil depletion studies have revealed a critical role for neutrophils in orchestrating post-infarction repair by influencing macrophage polarization toward a reparative phenotype (17), reinforcing the notion that an appropriately tempered inflammatory response is necessary after MI. Indeed blunt abrogation of integrin CD11/CD18 (18) and P-selectin (19), while presenting impressive results in experimental animal studies, failed to convincingly improve outcomes in clinical studies. This reinforces the need to develop a better understanding of MI pathogenesis, particularly in humans.

Shortly after neutrophil infiltration reaches a peak at ~24-h post-MI, monocytes are recruited to the site of injury and begin to take on a critical role in infarct wound healing and tissue repair (Figure 1A). The infiltration of monocytes is facilitated by an increased mobilization of monocytes into the blood from the bone marrow as well as from extramedullary tissue including the spleen, which has recently been recognized to serve as a monocyte reservoir that can be activated following injury (20). Early recruitment of monocytes to infarcted myocardium is primarily regulated via the monocyte chemoattractant protein (MCP)-1/chemokine receptor (CCR)-2 axis, as pro-inflammatory monocytes (e.g., Ly-6C^hi^ cells in mice) expressing high levels of CCR2 are attracted to injured tissue expressing the CCR2 ligand MCP-1 (21). As a result, the initial wave of monocyte infiltration, which peaks ~3 days after reperfused MI, is characterized by an influx of pro-inflammatory monocytes that differentiate into tissue macrophages and promote removal of necrotic debris and tissue digestion via release of proteolytic enzymes such as matrix metalloproteinases and cathepsins (22). Subsequently, this inflammatory response gives way to a reparative response mediated in large part by a shift in monocyte and macrophage function toward tissue repair through increased expression of anti-inflammatory, pro-fibrotic, and angiogenic growth factors such as interleukin (IL)-10, transforming growth factor (TGF)-beta, and vascular endothelial growth factor (VEGF) (5). Although it was initially proposed that this shift in monocyte/macrophage function was mediated by recruitment of reparative (e.g., Ly-6C^lo^) monocytes from the blood, recent work in mice has demonstrated that reparative macrophages are also derived from inflammatory (Ly-6C^hi^) monocytes that are recruited from the blood, undergo a phenotypic switch to anti-inflammatory (Ly-6C^lo^) macrophages, and proliferate within the infarct to resolve inflammation and promote wound healing (Figure 1B) (23).

As with neutrophils, the balanced, timely, and restrained infiltration of monocytes/macrophages is necessary for successful post-infarction healing, as macrophage depletion studies have demonstrated impaired healing and worsened cardiac function after MI (24, 25). However, experimental studies inducing excessive elevations in monocyte and/or macrophage numbers have shown impaired infarct healing and adverse ventricular remodeling as well (26, 27). Thus, therapeutic strategies to limit the supply of inflammatory monocytes after MI continue to receive attention, bolstered by experimental data demonstrating that interventions to reduce monocyte infiltration can reduce infarct size and improve post-infarction cardiac function in rodent models (7, 28). In this regard, while a number of attempts have been made to apply anti-adhesive selectin-ligand targeted therapies to augment post-MI repair and reduce ischemia-reperfusion injury, these have mostly failed to yield favorable results. The above discussion suggests that rather than completely blocking particular or all leukocyte populations, more nuanced strategies that modulate the levels of specific sub-populations in a timed manner may be more beneficial.

### Proliferative Phase of Post-infarction Repair

In addition to initiating the inflammatory phase of post-infarction repair, infiltrating leukocytes also play an important role in the timely suppression and spatial containment of inflammation to facilitate transition toward the proliferative phase of healing. This stage typically begins ~4–7 days after reperfusion and is characterized by resolution of inflammation and proliferation of fibroblasts to initiate the formation of a collagen-rich scar. Neutrophils that had infiltrated the injured area early after reperfusion undergo apoptosis and subsequent phagocytic uptake by macrophages, which induces a phenotypic switch toward a pro-resolving (i.e., “M2”) macrophage phenotype characterized by release of anti-inflammatory and pro-fibrotic cytokines including IL-10 and TGF-β (5). Furthermore, apoptotic neutrophils express scavenging chemokine and cytokine receptors that reduce tissue levels of pro-inflammatory mediators, further contributing to a shift toward an anti-inflammatory micro-environment (29, 30).

Beyond the macrophage phenotypic switch elicited by phagocytosis of apoptotic neutrophils, additional leukocyte, and lymphocyte populations contribute to the proliferative phase of repair. For example, CD11c^+^ dendritic cells infiltrate the infarcted myocardium during the proliferative phase of repair and contribute to resolution of inflammation, scar formation, and angiogenesis. These effects appear to be mediated via clearance of pro-inflammatory cell types, as experimental ablation of dendritic cells in a rodent model of MI has been shown to result in sustained expression of inflammatory cytokines, persistent infiltration of pro-inflammatory monocytes and macrophages, and deterioration of left ventricular function (31). Anti-inflammatory T-lymphocyte populations also infiltrate the infarct area during the proliferative phase of repair and facilitate the transition toward maturation. This includes CD4^+^ and CD8^+^ T-cells, regulatory T-cells, and natural killer T-cells that may be activated by as-yet-unknown cardiac autoantigens and limit adverse ventricular remodeling by promoting wound healing, inflammation resolution, and scar development via collagen matrix formation (32). Regulatory T-cells (CD4^+^Foxp3^+^) may be particularly important in this context: Weirather et al. recently used a model of genetic regulatory T-cell ablation and an anti-CD25 monoclonal antibody to demonstrate that this population of T-lymphocytes modulates monocyte/macrophage polarization, myofibroblast activation, and collagen expression within the developing infarct scar to encourage wound healing after MI (33). Furthermore, increasing regulatory T-cell activation with a superagonistic anti-CD28 monoclonal antibody administered 2 days after MI led to improved infarct healing and survival compared with untreated controls, suggesting that therapeutic activation of regulatory T-cells may be a promising approach to boost cardiac repair and limit adverse ventricular remodeling (34).

Fibroblast expansion and conversion to a synthetic myofibroblast phenotype is key component of the proliferative phase of post-infarction repair (Figure 1A). In this process, inactivated fibroblasts become activated and develop expression of contractile proteins including α-smooth muscle actin (35). Although myofibroblasts contribute to the inflammatory phase of repair via secretion of pro-inflammatory cytokines and matrix metalloproteinases, they take on a more central role in the proliferative phase by producing anti-inflammatory and pro-angiogenic factors that facilitate the formation of granulation tissue. The source of these cells remains incompletely understood, but it has been suggested that myofibroblasts arise from either resident fibroblasts (36) or circulating bone marrow progenitor cells (37). Additional possible sources of myofibroblasts in the infarct include endothelial cells (via endothelial-mesenchymal transition) and epicardial epithelial cells. Regardless of the source, acquisition of a myofibroblast phenotype leads to proliferative activity and synthesis of extracellular matrix proteins including collagen, fibrin, and fibronectin, all of which contribute to the early phases of scar formation.

The dynamic extracellular matrix changes occurring during the proliferative phase of infarct healing are driven in large part by the induction of matricellular proteins that primarily direct cytokine and growth factor responses rather than provide structural support. These matricellular proteins include thrombospondins, tenascins, periostin, osteopontin, osteoglycin, and proteins from the secreted protein acidic and cysteine-rich (SPARC) and CCN families (38). Along with other proteins from the galectin and syndecan families, these matricellular proteins modulate protease and growth factor activity to provide spatial and temporal regulation of several processes that characterize the transition between initial inflammatory activation and scar formation. For example, structural matrix assembly, angiogenesis, fibrinogenesis, growth factor signaling, and regulation of inflammation have all been demonstrated to be influenced by matricellular proteins (38). Furthermore, it has been suggested that the selective localization of matricellular protein expression in the infarct border zone plays an important role in localizing inflammatory and fibrotic responses to the site of injury, despite diffusion of secreted growth factors and cytokines to remote, non-infarcted tissue (38). As a result, inappropriate induction of matricellular proteins beyond the infarct border may contribute to infarct expansion and adverse ventricular remodeling, thereby representing a possible therapeutic target to enhance post-infarction repair. Similarly, expression of matricellular proteins for an extended period of time during the proliferative phase of healing may lead to excessive fibrosis following injury, as clearance of matricellular proteins is thought to be an important “stop” signal to limit pro-fibrotic signaling. While a vast majority of extracellular matrix proteins are glycosylation, very little is known regarding their role in regulating post-MI cell proliferation and repair.

### Maturation Phase of Post-infarction Repair

Following the proliferative phase of repair, the emerging scar undergoes a maturation process in which the extracellular matrix becomes cross-linked and reparative cells including myofibroblasts are deactivated, enter a quiescent state, and may undergo apoptosis (38). The precise mechanisms underlying myofibroblast deactivation and quiescence remain incompletely understood but likely involve withdrawal of pro-fibrotic growth factors and activation of inhibitory “stop” signals via matricellular protein clearance, as mentioned above. In addition, a time-dependent increase in the production of anti-fibrotic factors may diminish matrix synthesis and promote scar maturation. For example, interferon-γ-inducible protein-10 is known to be upregulated after MI and functions to prevent spatial expansion of the pro-fibrotic response beyond the infarct area via proteoglycan-mediated inhibition of fibroblast migration (39, 40).

Beyond scar maturation in the infarct area, late post-infarction remodeling is often characterized by inflammation and fibrosis in viable non-infarcted myocardium in remote areas of the heart (Figure 1B). This pattern of remodeling can lead to LV dilatation, global systolic dysfunction, and the onset of heart failure. Clinical studies demonstrate that this series of events is most common in patients with large infarcts as well as in those exhibiting greater initial inflammatory activation (41, 42). Local activation of macrophages and fibroblasts in the remote non-infarcted myocardium as a result of increased wall stress secondary to the loss of contractile activity of the infarct area is thought to occur in this scenario. Moreover, it has been suggested that incomplete or impaired resolution of myocardial inflammation in the late phases of repair may lead to amplification of post-MI injury over time and promote adverse LV remodeling (5). Alternatively, recent data support the intriguing possibility that a second wave of immune activation may occur, due in part to the structural remodeling of the spleen, heightened antigen processing, and trafficking of activated spleen-derived monocytes to the heart to promote apoptosis, fibrosis, and dysfunction (43). Further investigation of this area is necessary and may yield novel understanding of mechanisms underlying immune system-mediated remodeling of the heart in the late phases of post-infarction repair, ultimately leading to new treatments designed to inhibit this process and prevent the development of heart failure.

## Role of Selectins in Immune Cell Recruitment Following Ischemic Injury

Emerging evidence supports an important role for glycans in each of the phases of post-infarction repair described above, beginning with recruitment of circulating immune cells early after tissue injury. Leukocyte extravasation follows a sequential cascade of steps involving at least three sets of proteins. First, selectins expressed on the inflamed vascular endothelium (E- and P-selectin), leukocytes (L-selectin) and activated platelets (P-selectin). These are type-II transmembrane cell surface proteins with a calcium dependent C-type carbohydrate binding lectin domain that engage a diverse set of glycoproteins and glycolipids on the leukocyte surface. Second, chemokines expressed on the inflamed endothelium that bind their cognate receptors on leukocytes. Chemokine presentation and function is keenly regulated by the surface expression of glycosaminoglycans on the endothelium (44, 45). Third, integrins on the leukocytes that bind members of the immunoglobulin domain proteins (ICAM-1, VCAM-1) among other entities. Importantly, this binding activity is regulated by the N-linked glycosylation status of integrins (46, 47). Overall, leukocyte adhesion interactions to the vascular wall are regulated by all major families of cell surface carbohydrates: O- and N-linked glycans that are common to glycoproteins, carbohydrates attached to glycolipids and the extended glycosaminoglycan (48). While several excellent reviews in the field focus on the latter two aspects (45, 47), the focus of the current discussion is on the role of selectin-glycan interactions since this is a critical step that is necessary for immune cell recruitment.

**Figure 2:**
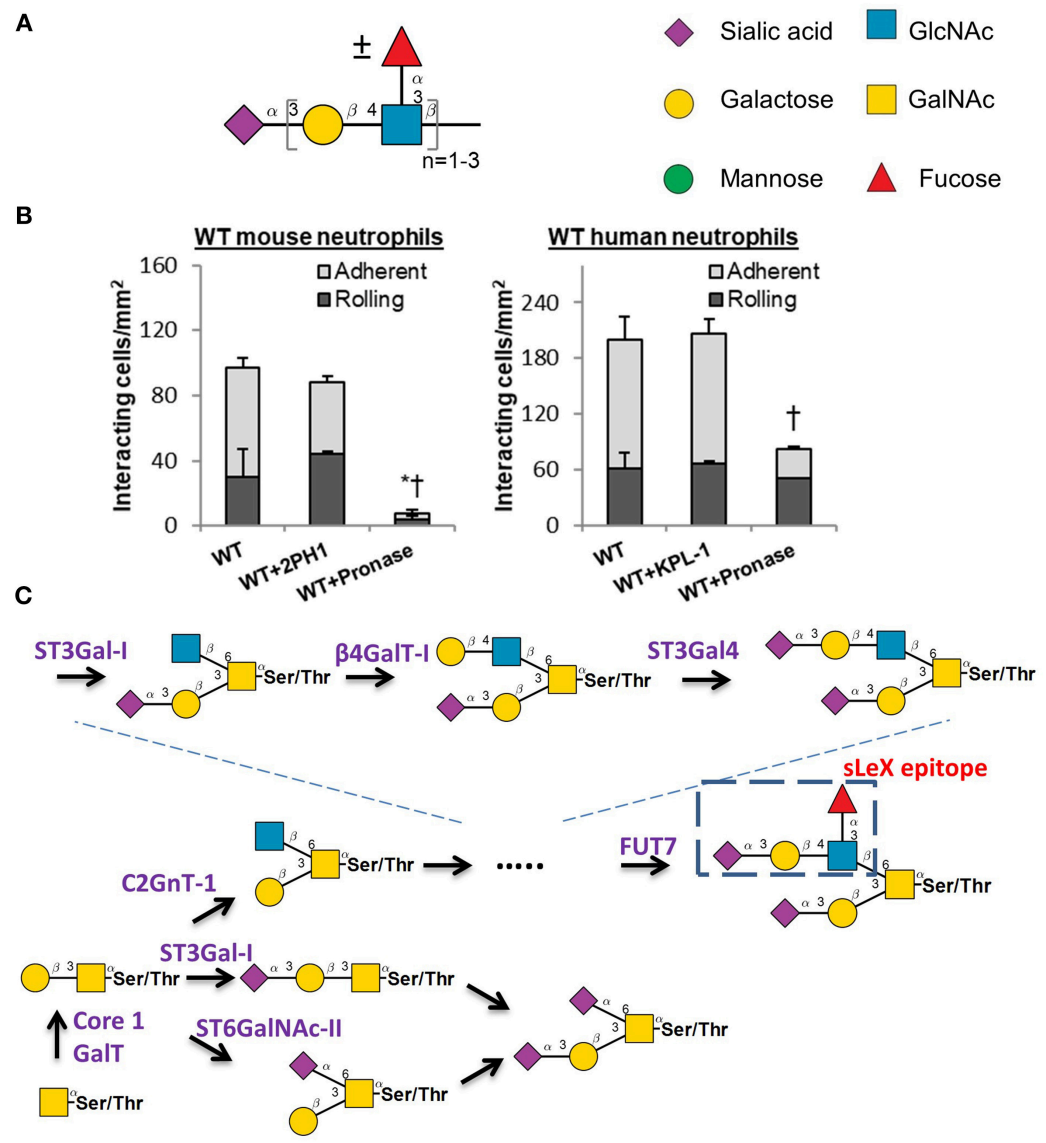
Selectin ligand biosynthesis. **(A)** Sialofucosylated selectin ligands found on leukocytes are composed of at least one α(2,3) sialic acid and one α(1,3) fucose, typically on a Type-II lactosamine chain that may repeat. **(B)** Human leukocyte rolling on HUVEC monolayer is resistant to pronase digestion, but this is not the case for mice. * and †: *P* < 0.05 for rolling and adherent cells, respectively. **(C)** Selectin ligand biosynthesis at the N-terminus of PSGL-1. Competing pathways regulate the biosynthesis of the sialyl Lewis-X (sLe^x^) epitope on core-2 based O-glycans. These competing enzymes are the core2 GlcNAc-transferase, ST3GaI-I, and ST6GaINAc enzymes. **(B)** is adapted from Mondal et al. (58) with permission.

Selectin-carbohydrate binding interactions are unique in mammalian physiology since the onset of this molecular interaction is rapid (high on-rate) and strong (high tensile bond strength) (49). These unique bond properties enable selectin-ligand binding to facilitate the rapid capture of flowing blood cells to sites of inflammation. The selectins bind sialofucosylated carbohydrate epitopes that typically bear one terminal α(2,3) sialic acid linkage and at least one α(1,3)-linked fucose attached to a type-II lactosamine chain (Galβ1,4GlcNAc). The prototypic selectin-ligand is called sialyl Lewis-X (sLe^X^, Neu5Acα2,3Galβ1,4[Fucα1,3]GlcNAc), though selected ligands can also appear on extended lactosamine chains that carry more than one α(1,3)fucose in a variety of configurations (50–52) (Figure 2A). Such selectin-ligands are generated commonly upon the post-translation modification of proteins at Ser/Thr or Asn sites, and to a lesser degree they may be found on specialized leukocyte surface glycolipids that are termed “myeloglycans” (53).

A variety of studies have been performed to identify the precise biosynthetic pathways that yield selectin-ligands and physiological selectin binding glycoproteins. These studies have primarily utilized mouse models (54). While this is generally a beneficial approach, there has been recent criticism that mouse models may have some important limitations with respect to their mimicry of human biology, particularly as it relates to the inflammatory response (55, 56). The development of newer RNA-interference (RNAi) technology and genome editing methods (CRISPR-Cas9) have led to studies that now utilize human leukemic cell lines and also primary human blood cells that are differentiated from CD34^+^ hematopoietic stem and progenitor cells (hHSPCs) for similar assays (57, 58). It has become evident in these studies that the adhesion molecules and enzyme-regulating selectin-ligand biosynthesis in humans and mouse are potentially organism-specific. This is most notably observed in studies that utilized pronase to cleave glycoproteins on the leukocyte cell surface, since mouse leukocytes fail to interact with stimulated endothelial cells following protease digestion, whereas the human counter-parts display robust cell adhesion under shear (58) (Figure 2B). This implies that at least some of the human selectin-ligands are protease insensitive, while this is not the case for mice. It is possible that these differences could in part, account for the failure of previous clinical trials that attempted to design anti-adhesion therapy for humans largely based on observations in murine models.

With regard to binding P-selectin, this adhesion molecule avidly binds an O-linked glycan that is located at the N-terminus of the leukocyte glycoprotein PSGL-1 (P-selectin glycoprotein ligand-1) (59). In humans, this glycan resides at Threonine 57 (T57) at the N-terminus of mature PSGL-1. The extended nature of P-selectin with 9 consensus repeat domains and the position of PSGL-1 at the tip of leukocyte microvilli enhance the probability that P-selectin will interact with its ligand under fluid shear (60). Thus, P-selectin binding to its ligand is often the first step that regulates leukocyte-endothelial cell adhesion interactions. It is now established that the O-glycan at the tip of PSGL-1 that binds selectins is a core-2 glycan with a terminal sLe^X^ structure (Figure 2C). The relative prevalence of this ligand is tightly controlled by the action of three competing enzymes that act to regulate core-2 structure biosynthesis: (i) Core-2 GlcNAc transferase (C2GnT-I) that forms this structure; (ii) ST6GalNAc enzymes that compete to add sialic acid at the 6-position of GalNAc (52), the same location as C2GnT-I; and (iii) The sialyltransferase ST3Gal-I which facilitates core 1 O-glycan sialylation, as its reduction promotes core 2 O-glycan biosynthesis (61) (Figure 2C). In this regard, it has been proposed that the balance between ST3Gal-1 and C2GnT-I plays a major role in controlling CD8^+^ T lymphocyte homeostasis. A dramatic shift from ST6GalNAc dominated α(2,6) sialylated structures to core-2 structures is also observed on T-cells as they transition from resting to activated states (62).

In addition to the above enzymes, studies using transgenic mice suggest additional glycoslytransferases that either partially or fully regulate sLe^X^ biosynthesis at the PSGL-1 N-terminus. These include polypeptide α-GalNAcT ppGalNAcT-1 (63), core-1 β1,3GalactosylT T-synthase (64), core-2 β1,6GlcNAcT C2GnT-I (65), β1,4GalactosylT β4GalT-I (66), α(2,3)sialylT ST3GalT-IV and VI (67, 68) and α(1,3) fucosyltansferases (FUTs), FUT7 (69), and FUT4 (70). Sulfation of the peptide backbone by tyrosine sulfotransferases is also important for functional selectin ligand biosynthesis on PSGL-1. The molecular players in human leukocytes is likely similar to mice in that FUT4 and FUT7 are the dominant contributors to L- and P-selectin binding under shear (71). ST3Gal-VI may however not be as significant in human leukocytes since knocking out ST3Gal4 (also called ST3Gal-IV) alone is sufficient to abolish cell rolling via both L- and P-selectin (57), both in studies performed with HL-60 cell lines and human neutrophils derived from CD34^+^ hHSPCs. The exact contributions of the other enzymes to human leukocyte adhesion remains unknown. Additionally, while it is reported that the CD16^−^CD14^+^ classical monocytes express higher sLe^X^ levels on the cell surface compared to non-classical CD16^+^CD14^dim^ (72), the relative contribution of C2GnT-I, ST6GalNAc enzymes in regulating the balance in monocytes is yet to be established, as much of the previous data were derived from neutrophils.

With regards to endothelial selectins, current consensus suggests that E-selectin is the dominant selectin in humans, while P-selectin may be dominant in mice. This is supported by observations that the promoter of the *Selp* (i.e., P-selectin) gene in mice, but not humans, has binding sites for multiple transcription factors including NF-κB and ATF-2 (73). Due to this, P-selectin is both secreted from storage granules and it is transcriptionally upregulated in mice upon stimulation with TNF-α, IL-1β, and LPS. In contrast, whereas P-selectin granule stores exist in humans and rapid exocytosis is noted upon inflammatory stimulus, longer term transcriptional control is absent. In support of this, following inflammatory TNF stimulation, wild-type mice exhibit slow leukocyte rolling and increased cell adhesion unlike transgenic human P-selectin expressing animals that display more rapid rolling and reduced adhesion (73). Overall, the basal and inducible levels of P-selectin differ across species, and its relative contribution to mouse leukocyte adhesion is higher compared to man. Thus, the perceived “central role” of P-selectin during inflammation based on murine studies may not hold in humans where E-selectin likely has a larger role (73, 74).

Besides the above difference in selectin expression patterns, current data suggest that the major E-selectin ligands identified in mice, ESL-1, CD44, and PSGL-1/CD162 (75), may not play a dominant role during human leukocyte rolling on either recombinant E-selectin or IL-1β stimulated human umbilical vein endothelial cells. In this regard, there is no homolog for ESL-1 in humans (76). PSGL-1 is a relatively minor E-selectin ligand in humans: anti-PSGL-1 blocking antibodies do not block human neutrophil binding to E-selectin (77, 78), and CRISPR-Cas9 knock-out HL-60s lacking PSGL-1 roll robustly on endothelial cells (HUVECs) under shear. Finally, while a specific glycoform of CD44 is known to act as an E-selectin ligand on hHSPCs, it has been previously reported to be absent in mature human leukocytes (75, 79, 80). In recent studies focused on the identification of E-selectin ligands on human monocytes, Silva et al. (72) demonstrate the expression of the E-selectin binding sLe^X^ epitopes on classical monocytic O-linked glycans expressed on CD43, CD44, and CD162; and possibly also one other yet unidentified 60–70 kDa glycoprotein. Further studies using genetic ablation and blocking mAbs are needed to confirm the potential role of these ligands in human monocyte recruitment to the injured heart. Interestingly, while the O-glycans of CD44 are reported to facilitate monocyte binding in this study, it is the CD44 N-glycans that facilitate E-selectin binding to T-cells. This suggests cell specific differences in glycosylation machinery even within leukocytes sub-populations of a single species.

Besides differences in the protein scaffolds, many studies from our laboratory show that the glycosyltransferases synthesizing E-selectin ligands may differ between humans and mice. Specifically, the α(1,3)fucosyltransferase FUT9 has a more significant role during human leukocyte adhesion (71), compared to FUT7 and FUT4 which are the dominant players in mice (69, 81). Knocking out the α(2,3)sialyltransferase (sialylT) ST3Gal-4 abrogates E-selectin binding (and also other selectins) in humans (57) but this is only partially effective in mice (68, 82). Knocking out glycosphingolipid (GSL) biosynthesis using CRISPR-Cas9 by targeting the enzyme UGCG (UDP-Glucose Ceramide Glucosyltransferase) results in skipping/unstable rolling motion of human myeloid cells on E-selectin (58).

**Figure 3:**
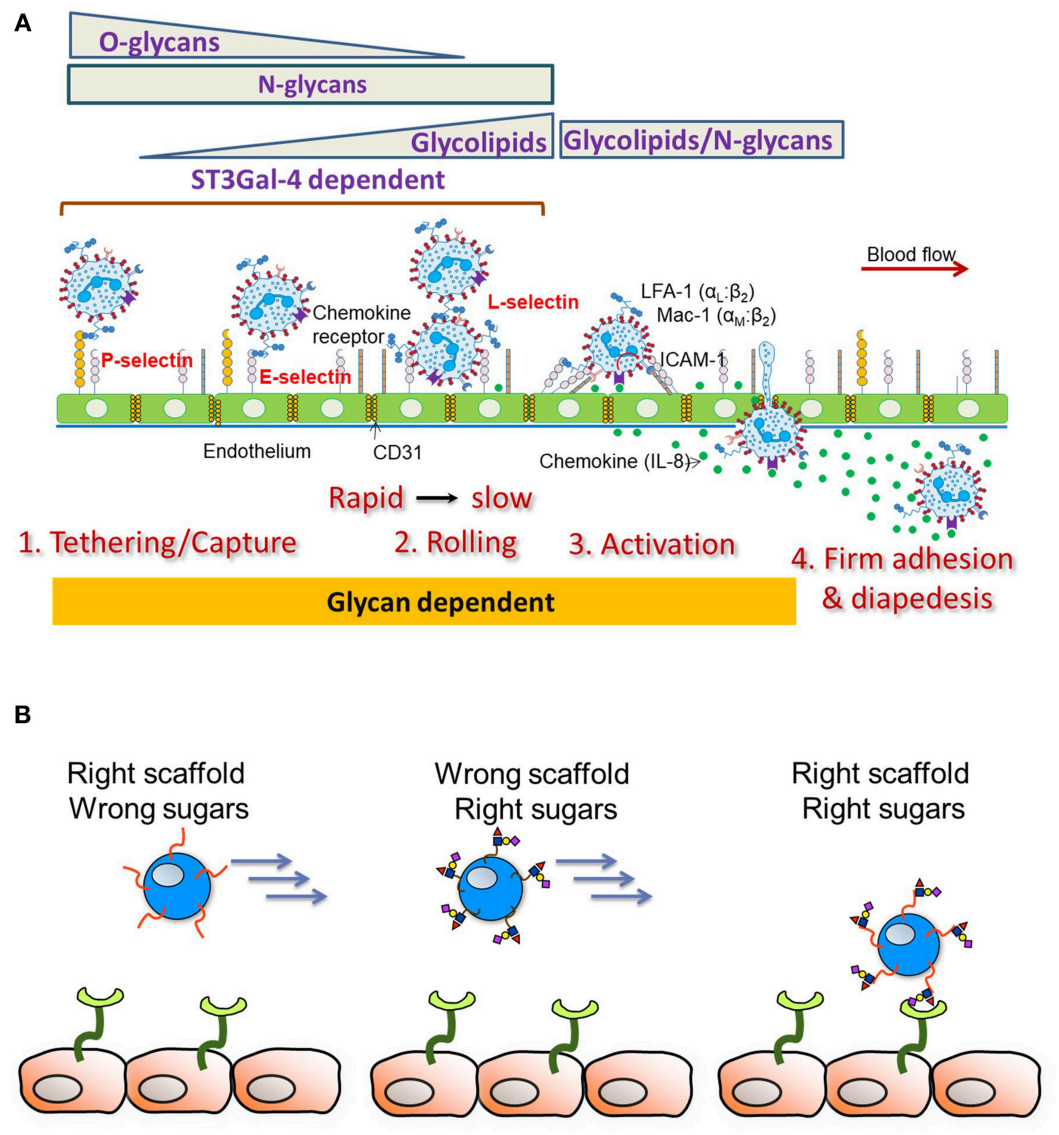
Leukocyte-endothelial cell adhesion cascade. **(A)** Schematic depicts results from studies using human cells. The sialyltransferase ST3GaI4 is indispensable for all aspects of leukocyte adhesion. O-glycans are important for leukocyte capture from flow along with N-glycans. N-glycans largely control leukocyte rolling velocity with glycolipids also contributing to the transition to firm arrest. Besides cell adhesion, emerging evidence points to a role for glycans in also regulating cell activation and the transition to firm arrest. **(B)** Endowing heterologous cell types (like Mesenchymal Stem Cells, MSCs) with selectin scaffold proteins (“right scaffold”) along with enzymes facilitating the construction of sialofucosylated glycans (“right sugars”) can enable stem cell capture and rolling on the inflamed endothelium.

In order to dissect the contributions of N-glycans, O-glycans and glycolipids on human leukocyte cell adhesion, Stolfa et al. (83), made a panel of 7 single, dual and triple knockout cell lines on the human leukemia HL-60 background that bear truncated glycoconjugates (Figure 3A). The investigators tested the ability of these cells to be recruited and to roll on recombinant E-selectin substrates and HUVECs. Their studies demonstrated that O- and N-linked glycans, both, control the initial recruitment of neutrophils from flow with N-glycan primarily regulating neutrophil rolling velocity. Whereas, glycolipids did not play a role in the initial recruitment, their proximity to the cell membrane allowed their participation in the slow rolling process, which eventually lead to firm arrest.

Overall, the expression pattern of scaffold proteins bearing the carbohydrate-ligands and the level of cellular glycosyltransferase activity are important parameters that define the E-selectin ligand. More detailed studies are needed to determine if the protein scaffolds and glycan structures identified above for human neutrophils, also contribute to the adhesion patterns of other immune cell types relevant to post-infarction repair. The identification of the E-selectin binding glycoconjugates and related blocking antibodies will greatly simplify this quest.

## Therapeutic Efficacy of Interventions Aimed at Interrupting Selectin-Mediated Immune Cell Infiltration After Myocardial Infarction

In light of the key role that selectins play in immune cell recruitment, adhesion, and tissue infiltration after ischemic injury, efforts to diminish the detrimental effects of pro-inflammatory immune cells have focused on interrupting selectin-mediated cell adhesion after MI. The earliest approaches centered upon the use of monoclonal antibodies directed against specific selectin molecules. After it was determined that P-, L-, and E-selectin each recognize the common ligand sLe^X^, carbohydrate sLe^X^ analogs were developed and tested as a soluble selectin blocker (84). In addition, discovery of PSGL-1 as a high-affinity ligand for P-selectin encouraged the development of soluble PSGL-1, which has been utilized for functional blockade of selectins *in vivo* (85). The following section summarizes progress that has been made over the past two decades in the investigation of anti-inflammatory therapies designed to interfere with selectin-immune cell interactions after myocardial ischemic injury.

P-selectin antagonism has been attempted using sLe^X^ and sPSGL-1 analogs in experimental studies of myocardial infarction. Initially, Buerke et al. found a significant, 83% reduction in myocardial infarct size following myocardial ischemia/reperfusion in cats that were treated with CY-1503, a sLe^X^ oligosaccharide (86). Importantly, the reduction in infarct size elicited by CY-1503 treatment also led to improved cardiac functional performance, based on invasive measurements of cardiac contractility (dP/dt_max_). Subsequently, Silver et al. tested CY-1503 in a large animal (dog) model and found a nearly 70% reduction in infarct size relative to the ischemic area-at-risk 1-h after reperfusion, with a marked reduction in myeloperoxidase activity compared with controls that was consistent with reduced neutrophil infiltration in CY-1503-treated animals (87). The duration of this benefit was extended to 48-h in a later study by Flynn et al., in which CY-1503 treatment produced ~55% reductions in both infarct size and neutrophil infiltration in a canine model of myocardial ischemia/reperfusion injury (88). Such studies with classical sLe^X^ analogs fell out of favor, partly due to the low binding affinity of such entities and limited circulatory half-life (49). Nevertheless, better design of sLe^X^ synthetic analogs have recently emerged, with compounds like GMI-1070 beginning late-stage clinical trials for sickle cell disease (89). It is possible that success in such orphan disease studies may pave the way for future trials related to myocardial infarction.

Following the discovery of the major P-selectin ligand PSGL-1, attention shifted toward analogs of this glycoprotein since the binding affinity of PSGL-1 for P-selectin is ~1,000 times that of sLe^X^ (90, 91), and mouse studies demonstrated an important role for murine PSGL-1 in leukocyte trafficking and neutrophil recruitment following inflammatory injury (92). Following initial positive studies in isolated rat hearts (93) and cats subjected to myocardial ischemia/reperfusion injury (94), efficacy of a recombinant soluble P-selectin glycoprotein ligand-Ig (rPSGL-Ig) was confirmed in a canine model of reperfused MI (95). In this study, dogs were subjected to 90-min of regional myocardial ischemia via balloon occlusion of the left anterior descending coronary artery. Fifteen minutes after reperfusion (achieved by balloon deflation), an intravenous bolus of rPSGL-Ig (1 mg/kg) or saline was administered. Animals were followed for either 2-h or 7-days, at which time infarct size, neutrophil infiltration, and myeloperoxidase activity were assessed. rPSGL-Ig-treated animals exhibited a significant reduction in infarct size relative to the ischemic area-at-risk compared with saline-treated animals at both time points, as well as diminished neutrophil infiltration and myeloperoxidase activity in the ischemic region of the left ventricle (95). These beneficial effects of rPSGL-Ig treatment were associated with an improvement in left ventricular ejection fraction 24-h after reperfusion, although both treatment groups exhibited improvements in ejection fraction over the following week and group differences were no longer statistically significant 7-days after reperfusion.

Besides selectin-ligand analogs, the therapeutic administration of monoclonal antibodies has largely focused on inhibition of P-selectin, based in large part on early experimental studies showing beneficial effects of anti-P-selectin antibodies in animal models of myocardial infarction. For example, administration of anti-P-selectin antibodies resulted in a ~60% reduction in myocardial infarct size and preserved coronary vascular endothelial integrity in a feline model of myocardial ischemia/reperfusion injury (96), a finding that was subsequently reproduced in a canine model (97). Translation of these findings to clinical testing eventually led to the completion of the SELECT-ACS trial, in which a highly specific human recombinant monoclonal antibody direct against P-selectin (inclacumab; 5 or 20 mg/kg) was administered to more than 200 patients undergoing percutaneous coronary intervention for non-ST-segment MI (NSTEMI) (19). It is important to note that the rationale for conducting this study was primarily based on the hypothesis that P-selectin antagonism would exert beneficial effects on peri-procedural coronary vascular injury by minimizing platelet adhesion, macrophage accumulation, and neointimal formation at the site of revascularization. Nevertheless, biomarkers of cardiac injury including cardiac troponin I and creatine kinase-myocardial band tended to be lower in patients treated with inclacumab (*p* = 0.05–0.10), suggesting that P-selectin antagonism has some benefit in NSTEMI patients. Interestingly, a follow-up subgroup analysis of the SELECT-ACS trial was performed and demonstrated that the beneficial effects of inclacumab were particularly pronounced in patients that received treatment <3-h before percutaneous coronary intervention (98). This finding reinforces the importance of understanding dynamic time-dependent changes in selectin expression after injury to allow effective therapeutic targeting, particularly when agents with a relatively short half-life are used.

Collectively, these results provide support for the notion that P-selectin blockade may offer therapeutic benefits after myocardial infarction, although strong clinical data are lacking. The reason that the encouraging preclinical results described above have not been rapidly translated to human patients is not immediately clear, although this generally parallels the overall experience to date with anti-inflammatory strategies to treat patients with MI (99). Furthermore, efforts to interrupt neutrophil infiltration after MI via the administration of antibodies against endothelial integrins such as CD18 and CD11 have been unsuccessful in clinical studies, which may have discouraged testing of selectin blockade strategies. Another possible issue relates to the short duration of follow-up that is often employed in preclinical studies. Because infarct size and inflammatory cell infiltration were typically measured in the first several hours after reperfused MI in initial experimental animal studies, the contribution of selectin-mediated immune cell recruitment at later stages of post-infarction repair may have been overlooked. Taken together with information described above indicating species-specific differences in the role of particular selectins in mediating immune cell recruitment and the distinct contributions of different leukocyte sub-types to the injury and repair process, it is possible that E-selectin has been overlooked as a potential therapeutic target. Indeed, early studies testing E-selectin antagonism with monoclonal antibodies did not observe a positive effect on infarct size when measured within the first 4-h after reperfusion, consistent with data demonstrating that coronary vascular expression of E-selectin is minimal during this early post-injury time frame (100). However, the evolution of our understanding of post-infarction healing has revealed that pathological and reparative processes contributing to ventricular remodeling after MI occur at stages beyond the initial hours following reperfusion. Thus, E-selectin antagonism targeted to the acute post-MI inflammatory phase (e.g., 0–24 h after reperfusion) may be an attractive therapeutic strategy as it would diminish neutrophil-mediated reperfusion injury without interfering with the subsequent monocyte-driven repair phase. Furthermore, E-selectin expression by endothelial cells in the bone marrow and spleen has recently been shown to regulate hematopoietic stem cell proliferation, as well as monocyte production and release into the blood after myocardial ischemic injury (101, 102). Further studies are therefore necessary to determine whether novel therapies targeting E-selectin, perhaps in combination with P-selectin blockade, may offer the dual benefit of dampening post-infarction inflammation via a two-pronged approach involving interruption of leukocyte mobilization from the bone marrow and spleen, as well as leukocyte extravasation at the site of myocardial injury. With the appropriate study design that allows for evaluation of efficacy beyond the first several hours after reperfusion, monoclonal antibodies, antisense oligodeoxynucleotides (103), and nanoparticle-based RNA interference-based approaches (104) could each be useful.

## Enhancing the Delivery of Stem Cell Therapeutics by Mimicking Natural Immune Cell Recruitment Mechanics

Because myocyte loss is a fundamental component of ischemic injury and adverse post-infarction remodeling, stem cell-based therapy has emerged as a promising approach to restore cardiac function after MI (105). However, progress in this field has been stymied due, at least in part, to challenges related to cell survival and engraftment after injection. Accordingly, there is interest in learning from the natural homing process of immune cells to inflamed myocardium and glycoengineering stem cells with selectin ligands and other features to enhance retention at sites of cardiac injury. Such cells exhibit low immunogenicity and express multiple bioactive compounds including chemokines and growth factors that may enhance cardiac repair and promote myocardial regeneration. Moving beyond whole cells, stem cell-derived exosomes and microvesicles can also be targeted to deliver microRNA and proteins for therapeutic benefit. These vesicles often contain pro-angiogenic and pro-fibrotic factors that promote endothelial proliferation and TGF-β driven repair processes (106). Such modified cells and exosomes may be administered via a variety of delivery methods including intra-venous (i.v.), intra-arterial (i.a.), or intra-coronary (i.c.) infusion in order to enable their targeting to sites of injury. With respect to the mode of infusion, i.c. injection may be the most suitable approach since i.v. injection results in the delivery of all cells to the right-side of the heart with possible trapping and retention in non-targeted lung alveolar capillaries (107). Arterial injection is also an alternate approach that may result in both directed and passive entrapment within arterial microvasculature. In this regard, stem cell type (e.g., typical mesenchymal stem cells/MSCs and cardiosphere derived stem cells/CDCs) are typically 1.5–2-fold larger than normal blood cells, and they are mechanically more rigid. The larger size increases the drag force applied on the cells under fluid shear by a factor of 2–4 compared to blood leukocytes, thus making it more challenging to capture these cells in large vessels (108). However, this enhanced size makes them more prone to entrapment in the microcirculation, a region where endothelial cells highly expresses adhesion molecules relevant to inflammation and injury.

An advantage of systemic injection is that unlike localized injection at focal regions of damage that often have reduced nutrient and oxygen levels and suffer from risks of tissue perforation, systemic infusion may allow targeting of the cellular therapeutic in a less invasive manner. Ideally, such cells would be delivered to a well-vascularized, border regions of the heart that is viable, but compromised due to damage to the surrounding tissue. Such regions are often inflamed and they express high levels of selectins, chemokines, and integrins. While early studies suggested that the mesenchymal stem cells (MSCs) may constitutively express selectin and integrin ligands, it is now believed that such expression is not robust, and is highly dependent on the nature of *in-vitro* propagation conditions (109). Additionally, MSCs are not a uniform cell type as surface markers vary between sources and with passage number. Based on this, it is currently thought that the artificial over-expression of selectin-ligand is necessary on stem cells as this may improve targeted delivery by mimicking blood neutrophils that have high tropism for sites of vessel injury. This may then reduce the number of stem cells required for therapy and minimize off-target effects, enhancing retention in the heart above the ~1–2% of injected dose that is common when the cell surface is unmodified (110).

In support of this concept, Xia et al. (111) demonstrated that human umbilical cord blood CD34^+^ cells contain reduced amounts of sLe^X^ expression. The over-expression of this sialofucosylated epitope upon addition of recombinant fucosyltranferase enzyme FUT6 along with GDP-fucose (guanosine diphosphate-fucose) donor enabled the assembly of robust levels of the sLe^X^ epitope on the cell surface. Enhanced CD34^+^ stem cell rolling on endothelial monolayers expressing P- and E-selectin ensued and transplantation of these modified cells enhanced blood cell engraftment in mouse models. Similar to this, recent pilot first-in-human trials also suggest that this approach may be feasible clinically as it led to faster neutrophil and platelet engraftment (112). Similar to cord blood, MSCs can also be engineered to overexpress α(1,3)fucosyled epitopes via exofucosylation (109) and also using modified RNA (113) to enhance cellular targeting particularly to the bone, since the marrow constitutively expresses E-selectin. In all these studies, the precise glycoproteins that act as functional selectin-ligands is not fully established though there are suggestions that a sialofucosylated glycoform of CD44 called HCELL may be a key player (109).

An understanding of the precise E-selectin ligand on human leukocytes and also α(1,3)fucose modified stem cells is critical since neither the scaffold protein itself nor the sLe^X^ epitope alone can mediate robust stem cell recruitment under fluid flow conditions (Figure 3B). In agreement with this, when Lo et al. (114) modified MSC and also CDC cell surfaces to express an N-terminus PSGL-1 glycopeptide on a fusion protein scaffold, robust cell rolling interactions on P-selectin was only observed when the PSGL-1 O-linked glycan contained a core-2 sLe^X^ structure. In the absence of either the PSGL-1 protein scaffold or the sLe^X^ glycan, stem cell interaction with the selectin substrate was absent. In contrast to site-specific α(1,3)fucosylation on the PSGL-1 glycoprotein, when global α(1,3)fucosylation was performed on all stem cell glycoconjugates, robust leukocyte interactions were only observed on E-selectin substrates, but not P-selectin (115). The combined use of the glycosylated PSGL-1 glycoproteoform along with global α(1,3)fucosylation was needed for the robust binding of stem cells on all selectin substrates and also on endothelial cell monolayers in microfluidics flow studies. Such glycoengineering of stem cells using the combined coupling strategies also enabled short-term retention of stem cells in the left anterior descending artery of the pig heart in a brief ischemia-reperfusion model (115). This study confirmed the safety of the cellular therapy in a pre-clinical large animal model. Besides, glycoengineering approaches which are aimed to recruit stem cells from flow, it has also been demonstrated that decorating hematopoietic stem cells with bispecific antibodies that bind human CD45 and myosin light chain, can enhance cell homing to infarcted myocardium (116). Overall, while there is preliminary data that targeting to the heart is feasible, more investigation is needed in order to determine if this leads to better clinical outcomes.

Besides the selectin-ligands, the overexpression of a variety of chemokines have also been shown to enhance stem cell homing and retention. These methods may enhance the delivery of endogenous chemokine and growth factor receptors to sites of inflammation and injury, thus aiding the activation of cell surface integrins and cellular homing response (110). In this regard, the over-expression of signaling processes via the CXCR4/CXCL12 axis in MSCs has been shown to enhance myocyte preservation in the infarct zone, possibly accompanied by enhanced engraftment (117, 118). In addition, the over-expression of the CCR-1 chemokine receptor on MSCs and direct cardiac injection have has been shown to enhance cardiac engraftment leading to reduced LV remodeling and enhanced recovery of function (119). Besides these, the incorporation of metalloproteinases (MMP-2 and MT1-MMP) during stem cell delivery may also help degrade extracellular matrix components and enhance stem cell migration to sites of injury.

## Conclusion

At the current time, there is a paucity of therapeutic approaches that target the immune system’s response to myocardial ischemic injury to favorably influence cardiac healing and repair. While early studies attempted to address this problem using broad anti-adhesive therapies, often by targeting sLe^X^-selectin binding, these were largely met with failure in human clinical trials. The failures may be in part due to an incomplete understanding of the role of leukocytes in cardiac repair, as recent studies show that this is a complex process orchestrated by numerous sub-families of white blood cells. Moreover, reparative monocytes and macrophages are necessary to improve cardiac function, and thus the blockade of all immune cells using blunt anti-adhesive therapies may have detrimental effects. In addition, recent studies highlight the need for more glycoscience-based investigation, to identify putative human E-selectin ligands in the leukocyte sub-populations and stem cells, as well as to clearly define biosynthetic checkpoints regulating selectin-ligand biosynthesis. There is also clear evidence now that the cellular metabolism which regulates selectin-ligand/glycan biosynthesis may differ between humans and other species like mice. Thus, precise species-specific differences need to be understood and systems-based perturbations are necessary to evaluate the detailed consequences of specific interventions in complex systems, prior to initiation of human trials. Such understanding can help the design of better anti-adhesive therapies, and ultimately reduce the high morbidity and mortality associated with ischemic heart disease by reducing excessive inflammatory injury and/or improving delivery of novel biological therapeutics to the heart.

## Author Contributions

All authors listed have made a substantial, direct and intellectual contribution to the work, and approved it for publication.

### Conflict of Interest Statement

The authors declare that the research was conducted in the absence of any commercial or financial relationships that could be construed as a potential conflict of interest.
